# A Novel Optical Intracellular Imaging Approach for Potassium Dynamics in Astrocytes

**DOI:** 10.1371/journal.pone.0109243

**Published:** 2014-10-02

**Authors:** Theresa S. Rimmele, Jean-Yves Chatton

**Affiliations:** 1 Department of Fundamental Neurosciences, University of Lausanne, Lausanne, Switzerland; 2 Cellular Imaging Facility, University of Lausanne, Lausanne, Switzerland; Albany Medical College, United States of America

## Abstract

Astrocytes fulfill a central role in regulating K^+^ and glutamate, both released by neurons into the extracellular space during activity. Glial glutamate uptake is a secondary active process that involves the influx of three Na^+^ ions and one proton and the efflux of one K^+^ ion. Thus, intracellular K^+^ concentration ([K^+^]_i_) is potentially influenced both by extracellular K^+^ concentration ([K^+^]_o_) fluctuations and glutamate transport in astrocytes. We evaluated the impact of these K^+^ ion movements on [K^+^]_i_ in primary mouse astrocytes by microspectrofluorimetry. We established a new noninvasive and reliable approach to monitor and quantify [K^+^]_i_ using the recently developed K^+^ sensitive fluorescent indicator Asante Potassium Green-1 (APG-1). An *in situ* calibration procedure enabled us to estimate the resting [K^+^]_i_ at 133±1 mM. We first investigated the dependency of [K^+^]_i_ levels on [K^+^]_o_. We found that [K^+^]_i_ followed [K^+^]_o_ changes nearly proportionally in the range 3–10 mM, which is consistent with previously reported microelectrode measurements of intracellular K^+^ concentration changes in astrocytes. We then found that glutamate superfusion caused a reversible drop of [K^+^]_i_ that depended on the glutamate concentration with an apparent EC_50_ of 11.1±1.4 µM, corresponding to the affinity of astrocyte glutamate transporters. The amplitude of the [K^+^]_i_ drop was found to be 2.3±0.1 mM for 200 µM glutamate applications. Overall, this study shows that the fluorescent K^+^ indicator APG-1 is a powerful new tool for addressing important questions regarding fine [K^+^]_i_ regulation with excellent spatial resolution.

## Introduction

The gradient of potassium ions across the membrane of mammalian cells, along with that of Na^+^, plays a vital role both in establishing the standing electrical potential and in powering numerous transport systems. In astrocytes, these gradients are critical not only for the maintenance of a cell's own internal homeostasis —such as the regulation of internal pH and ion composition— but they also enable the dynamic regulation of the brain interstitial milieu. Of particular importance, astrocytes play a predominant role in the clearance of neurotransmitters released in the synaptic cleft by neurons during synaptic activity [1]. They are also actively involved in taking up extracellular K^+^ ([K^+^]_o_) released by neurons during activity, particularly during the action potential repolarization phase [2]. Without adequate regulation, the increase in [K^+^]_o_ following action potentials would interfere with normal electrical activity and rapidly abolish network activity [2], [3]. Astrocyte express both active and passive mechanisms for K^+^ uptake and release, such as the inwardly rectifying potassium channel 4.1 (K_ir_ 4.1) [2], [4], the Na^+^/K^+^/Cl^−^ cotransporter, and the Na^+^/K^+^ ATPase [5], [6], and together these provide a highly efficient mechanism for rapid regulation of K^+^ by astrocytes in the brain. As a consequence of being extensively interconnected by gap junctions, astrocytes are capable of redistributing K^+^ to distant sites through the astrocytic syncytium [2].

Intracellular Na^+^ has been shown to undergo sizeable changes during activity [7]. This Na^+^ influx is caused in particular by the Na^+^-dependent uptake of glutamate, which has been identified to play a key role in the neuron-glia metabolic communication [8]. Glial glutamate uptake accomplished through as a secondary active process that involves the influx of three Na^+^ ions and one proton and the efflux of one K^+^ ion [9]–[11]. Therefore, intracellular K^+^ can be expected to undergo variations due to glutamate transport activity.

Thus, [K^+^]_i_ is potentially influenced both by [K^+^]_o_ fluctuations and by glutamate transport activity in astrocytes. It has been shown that K^+^ accumulates in astrocytes in response to increases in [K^+^]_o_ in slices and in cultured astrocytes of several species [12]–[16]. However, little is known regarding whether and how the intracellular K^+^ concentration is influenced by the transmembrane ion fluxes associated with glutamate uptake in astrocytes.

Ion-selective electrodes have been extensively used for extracellular K^+^ measurements (see e.g. [17], [18]). They have also been used for intracellular measurements of K^+^ concentration in the studies referenced above [12]–[16]. However, these approaches are relatively invasive and do not provide direct spatial information about K^+^ changes. Fluorescence-based detection of [K^+^]_i_ offers the benefits of being both non-invasive and capable of assessing the spatiotemporal pattern of K^+^ flux within the tissue. This approach, however, has not been straightforward to implement. The only available fluorescent dye to date, the ultraviolet probe Potassium-binding benzofuran isophthalate *(*PBFI) is difficult to load intracellularly, displays weak fluorescence, and has a K_d_ for K^+^ of ∼8 mM [19] that is far from the expected [K^+^]_i_ range, which is normally>100 mM. For these reasons, only a few reports have demonstrated successful use of this probe (see e.g. [17], [20], [21]).

The aims of the present study were (1) to characterize a novel K^+^-sensitive indicator named Asante Potassium Green-1 (www.teflabs.com) for its potential use as noninvasive and reliable tool to monitor and quantify [K^+^]_i_ during glial activity; (2) to employ this tool to investigate how [K^+^]_i_ changes in response to [K^+^]_o_ fluctuations and to glutamate application in primary mouse astrocytes. This new fluorescent indicator enabled us to demonstrate that primary mouse astrocytes display significant [K^+^]_i_ changes in the order of several millimolar both in response to [K^+^]_o_ level changes and to glutamate transporter activation. These changes likely play an important role in the integrated functions of astrocytes that are involved both in [K^+^]_o_ and extracellular glutamate regulation.

## Materials and Methods

### Cell culture

Every effort was made to minimize suffering and the number of animals used in all experiments. All experimental procedures were carried out in strict accordance with the recommendations of the Swiss Ordinance on Animal Experimentation and were specifically approved for this study by the Veterinary Affairs Office of the Canton of Vaud, Switzerland (authorization number 1288.5). Primary cultures of cortical astrocytes were prepared from 1 to 3-days-old C57Bl6 mice as described elsewhere [22]. Astrocytes were cultured for 3–4 weeks in DME medium (Sigma) supplemented with 10% FCS before experiments and were plated on glass coverslips for imaging.

### Spectrofluorimetry

The free acid form of the dye Asante Potassium Green-1 (APG-1) TMA salt or APG-2 TMA salt were used for the *in vitro* characterization. Fluorimetric analysis was performed in quartz cuvettes using a Perkin Elmer model LS55 luminescence/fluorescence spectrometer. Intracellular-like solutions were used for titrations and contained (mM): 12 NaCl, 5 MgCl2, 0.5 CaCl_2_, 1 EGTA, 10 HEPES, adjusted to pH 7.2 using N-methyl-D-gluconate. The K^+^ titration of the dye was obtained by successive additions of known amounts of K^+^-gluconate. The K^+^ selectivity of APG-1 over Na^+^ was analyzed using solutions containing (mM): 135 K^+^ gluconate, 5 MgCl_2_, 0.5 CaCl_2_, 1 EGTA, 10 HEPES, adjusted to pH 7.2 using N-methyl-D-glucamine, and increasing amounts of NaCl were added. For each K^+^ or Na^+^ concentration, emission spectra were recorded. Intracellular excitation spectra were recorded in living cells using a monochromator coupled to a Xenon arc lamp (Polychrome II, Till Photonics, Planegg, Germany) attached to an inverted fluorescence microscope (*see below*). Emission spectra following excitation at 488 nm were obtained using a LSM710 confocal microscope equipped with spectral detector (Carl Zeiss).

### Fluorescence microscopy

Dye-loaded cells were placed in a thermostated chamber designed for the rapid exchange of perfusion solutions and observation using oil-immersion objectives [23]. Cells were superfused at 35°C. Low-light level fluorescence imaging was performed on an inverted epifluorescence microscope (Axiovert 100 M, Carl Zeiss) using a 40×1.3 N.A. oil-immersion objective lens. Fluorescence excitation wavelengths were selected using a monochromator (Till Photonics) and fluorescence was detected using a 12-bit cooled CCD camera (Princeton Instruments) or EM-CCD camera (Andor). Image acquisition was computer-controlled using the software Metafluor (Universal Imaging, Reading, PA). Images were acquired at 0.2 to 1 Hz.

Experimental solutions contained (mM) NaCl, 137.4; KCl, 5.4; NaHCO_3_, 25; CaCl_2_, 1.3; MgSO_4_, 0.8; and NaH_2_PO_4_, 0.78, glucose, 5, bubbled with 5% CO_2_/95% air. When using different K^+^ concentrations, NaCl was adjusted to maintain isotonicity. Solutions for dye loading contained (mM) NaCl, 160; KCl, 5.4; HEPES, 20; CaCl_2_, 1.3; MgSO_4_, 0.8; NaH_2_PO_4_, 0.78; glucose, 20 and were supplemented with 0.1% Pluronic F-127 (Molecular Probes, Eugene, OR). In experiments involving more than one solution application, the order was alternated in order to exclude order-related effects. In this study, the standard reference [K^+^]_o_ of 5.4 mM was chosen to enable direct comparison with previous studies from our and other laboratories in the field of astrocyte research [23]–[25].

[K^+^]_i_ was monitored by loading astrocytes at 37°C for 40 min with the acetoxymethyl ester membrane permeant form of the dye APG-1 AM (12 µM) as described in [26]. APG-1 fluorescence was excited at 515 nm and detected at 535-585 nm (Chroma, Rockingham, VT). A similar *in situ* calibration procedure as previously described in mesangial cells and cerebellar granule cells was used as previously described [20], [27]. Briefly, astrocytes were permeabilized using 10 µg/ml nigericin and 10 µM valinomycin with simultaneous inhibition the Na^+^/K^+^-ATPase using 1 mM ouabain. Cells were then sequentially perfused with solutions buffered at pH 7.2 with 20 mM HEPES and containing 160, 140, 120 and 100 mM K^+^, respectively, and 30 mM Cl^−^, 135 mM gluconate with a constant total concentration of Li^+^ and K^+^ of 160 mM, replacing K^+^ by Li^+^ in the solution and keeping constant Na^+^ and Cl^−^ concentrations. The *in situ* calibration procedure was performed for each cell at the end of experiments where the K^+^ concentration is displayed in the graph ordinate, as described in previous studies on intracellular Na^+^ or pH (see e.g. [23], [28]).

Experiments using Sodium-binding benzofuran isophthalate (SBFI-AM, 15 µM, excited at 340 nm and 380 nm) or Fura-2 AM (8 µM, excited at 360 nM) together with APG-1 AM (excited at 490 nm) were performed using similar protocol as previously described [29]. For these experiments, the emitted fluorescence was detected through a 535 nm (35-nm bandpass) interference filter (Omega Optical, Brattleboro, VT).

For experiments requiring local delivery of K^+^, dye-loaded cells were placed in a customized open chamber enabling rapid exchange of perfusion solutions. A glass pipette of tip diameter 1–2 µm was filled with 500 mM K^+^-gluconate, approached using a micromanipulator (Sutter Instruments) at a distance of ∼10 µm from the cells. Pressure pulses of 200–500 ms, 1–2 psi were applied to the back of the pipette using a Picospritzer III (Sensortechnics, Puchheim, Germany) to locally deliver K^+^.

### Data analysis and statistics

Fluorescence intensity traces were drawn from regions of interest selected in up to 10 individual cells from the field of view using the Metafluor software package. Further calculations were done with Excel (Microsoft). Graphs and curve fitting were done with Kaleidagraph (Synergy Software, Reading, PA, USA). Unless otherwise indicated, a paired Student's t test was performed for each experimental group to assess the statistical significance against respective controls, *, **, and *** refer to p values of 0.05, 0.01, and 0.001, respectively. For estimation of EC_50_ values, non-linear curve fitting was performed using the Levenberg-Marquardt algorithm implemented in the Kaleidagraph software package. The equation used for fitting the dose-response analysis experiments is the following:
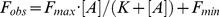
(1)were *F_obs_*, is the observed response, *F_max_, F_min_* are maximum, minimum parameters of the response. *[A]* is the concentration of agonist and *K* is the agonist concentrations that yield half-maximum responses (*i.e.* EC_50_). Equation [1] corresponds to a one-site Michaelis-Menten model.

### Dyes and drugs

Nigericin was from Abcam (Cambridge, UK), valinomycin from Fluka (Buchs, Switzerland). All other chemicals were from Sigma-Aldrich (Buchs, Switzerland).

## Results

### APG-1 characterization

**Figure 1:**
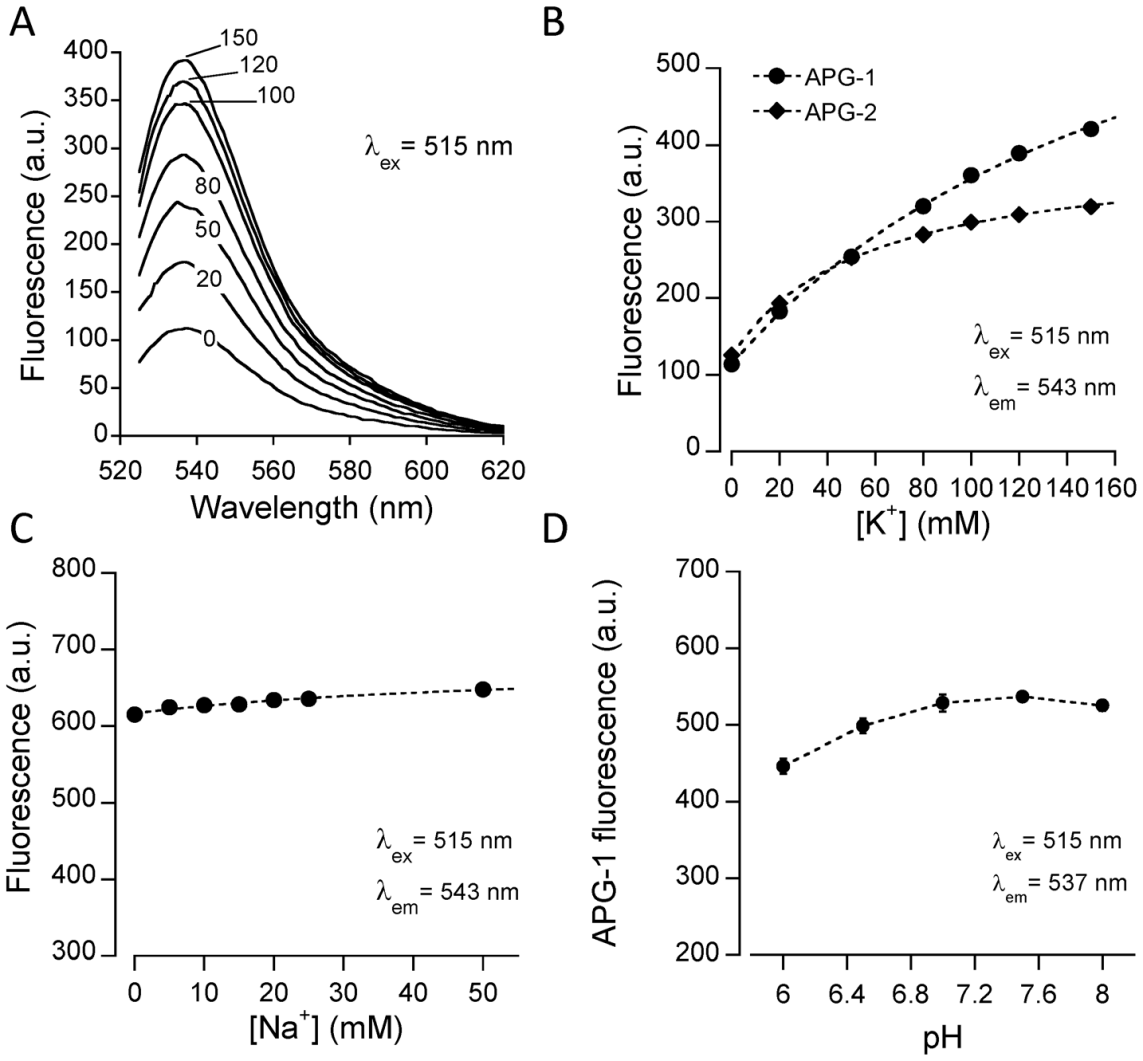
Spectrofluorimetric characterization of the K^+^ indicator APG-1. (**A**) Emission spectra recorded in the presence of different [K^+^] in intracellular-like solutions following excitation at 515 nm. Emission maximum was ∼540 nm. (**B**) Fluorescence emission plotted as a function of [K^+^] showing a monotonic relationship of APG-1 fluorescence with increasing [K^+^] (circles). The same analysis was performed on APG-2, a related indicator with identical spectral properties (diamonds) but lower Kd for K^+^. The plots show that APG-2 fluorescence becomes saturated at [K^+^]>80 mM, which is not the case with APG-1. (**C**) Na^+^ dependency of APG-1 fluorescence measured in intracellular-like solution containing 135 mM K^+^ (see also Fig. S1). (**D**) pH dependency of APG-1 fluorescence measured in intracellular-like solution containing 135 mM K^+^. The pH of each solution was adjusted using NMDG. This pH analysis was repeated three times. Data are presented as means ± SEM of triplicate measurements.

In a first phase, the K^+^ sensitivity and selectivity of the APG-1 fluorescent indicator was characterized *in vitro* using spectrofluorimetry. APG-1 is based on a similar design as the very successful sodium version Asante Natrium Green-1 [26]. For this purpose, APG-1 was dissolved in solutions corresponding to intracellular ionic composition, *i.e.* containing 10 mM Na^+^, nanomolar Ca^2+^ and millimolar Mg^2+^ concentration at pH 7.2. Excitation spectra presented a peak at ∼515 nm (*not shown*), whereas emission spectra had a maximum at ∼540 nm (**Fig.1A**), thus exhibiting spectral properties very similar to the related Na^+^ indicator Asante Natrium Green-1 [26]. Changing K^+^ concentration from 0 to 150 mM caused stepwise increases in fluorescence emission. APG-1 fluorescence emission versus K^+^ concentration (**Fig. 1B**) displayed a monotonic increase up to the expected upper physiological K^+^ concentration range of 150 mM. Neither the excitation nor the emission spectra displayed a shift in their maxima with increasing K^+^ concentration. The K^+^-sensitivity of the related indicator APG-2 was analyzed in parallel experiments. **Fig. 1B** shows that APG-2, as indicated by the manufacturer, has a higher affinity for K^+^ in identical intracellular-like solutions. APG-2 fluorescence reached saturation at K^+^ concentrations higher than ∼80 mM. This result indicates that APG-2 is unlikely to respond to K^+^ changes at physiological intracellular concentrations. Our tests performed with intracellular APG-2 (loaded using its membrane permeant form) confirmed this prediction, as no change or very small fluorescence changes were observable during cell stimulations such as by glutamate applications (*not shown*).

We next tested for potential influence of Na^+^, which is known to undergo rapid and substantial amplitude changes in astrocytes in the range 10–30 mM [7], [23], [30], in particular during glutamate transporter activity. **Fig. 1C** shows that Na^+^ concentrations up to 50 mM had only marginal influence on APG-1 fluorescence. **Fig. S1** extends this analysis and shows the effect of Na^+^ on APG-1 for different K^+^ concentrations ranging from 0 to 150 mM. It was found that in the absence of K^+^ the indicator displayed an expected increased sensitivity to Na^+^. However, for K^+^ concentrations within the range of intracellular levels ([K^+^]>100 mM), the interaction with Na^+^ was found to be minimal.

APG-1 is a weak acid, like other related fluorescent indicators of cations, and therefore it is potentially influenced by pH. We therefore tested whether pH had an influence on APG-1 fluorescence. **Fig. 1D** shows that the fluorescence of APG-1 was stable within the pH range of 7–8, found *in vivo* in brain cells under normal conditions. The figure also shows however that APG-1 fluorescence tends to decline below pH∼6.8, which should be taken into account in experiments involving substantial cellular acidification.

In summary, APG-1 is a fluorescent indicator in the visible light spectrum that displays in cuvette properties that make it very promising for intracellular K^+^ concentration measurements.

**Figure 2:**
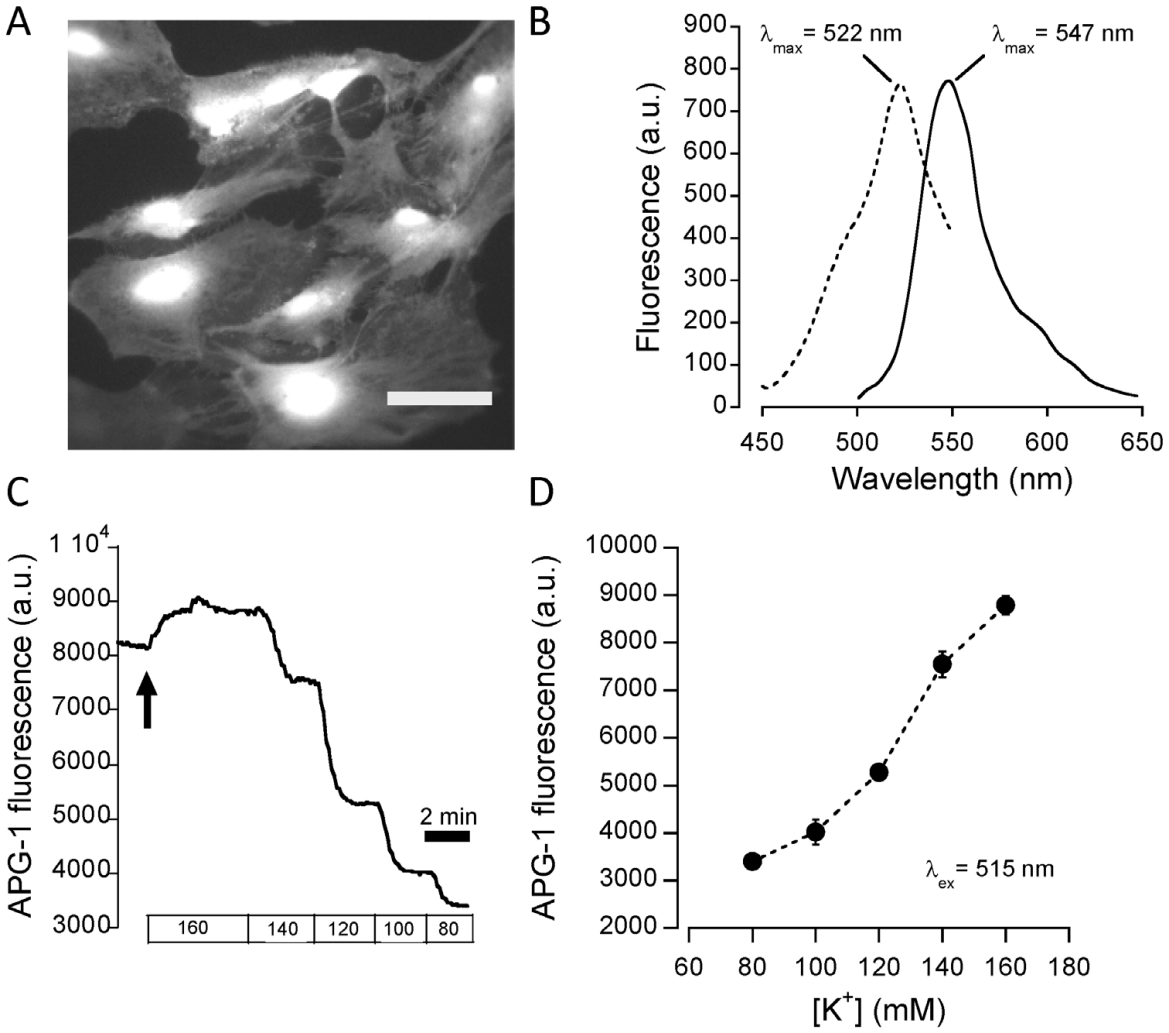
Intracellular characterization of the K^+^ indicator APG-1. (**A**) Fluorescence image of primary astrocytes loaded using APG-1 AM. Scale bar 50 µm. (**B**) *In situ* excitation and emission spectra measured by fluorescence microscopy. Intracellular spectra were ∼10 nm red-shifted compared with measurement in cuvettes. (**C**) Representative experimental trace depicting the *in situ* calibration procedure. At the time indicated by the arrow, the cell membrane was permeabilized for K^+^ using valinomycin and nigericin while the Na^+^/K^+^ ATPase was inhibited by ouabain. Solutions of different [K^+^] were then sequentially applied until stable fluorescence plateaus were obtained. (**D**) Calibration curve obtained by plotting the fluorescence plateau values measured for each known [K^+^].

In the next series of experiments, astrocytes were then loaded using the membrane permeant form of the dye, APG-1 AM. Cultured astrocytes adopt an extremely flat morphology (cell thickness typically∼1–2 µm) with the nucleus being the thickest part of the cell (typically 5–8 µm). When loaded with indicators with cytosolic localization that have the ability to permeate through nuclear pores, wide-field images of astrocytes show bright fluorescent nuclei, as they correspond to the cell region with the largest optical path length. As seen in **Fig. 2A**, cellular loading with APG-1 was strong and exhibited a typical of a cytosolic dye distribution. This property of APG-1 AM contrasts with the poor loading efficiency usually reported for the other K^+^-sensitive dye PBFI AM. Excitation and emission spectra of intracellular APG-1 were then recorded and showed maxima at 522 nm and 547 nm, respectively (**Fig. 2B**). Thus, *in situ*, the dye displays a slight red-shift of its spectra compared with in cuvette measurements. The probe exhibited a remarkable intracellular stability, with negligible photobleaching or cell leakage, as observed previously with the related sodium probe ANG-1 [26]. Next, an *in situ* calibration procedure was developed and optimized, applying general principles used for calibration of Na^+^ indicators. After permeabilization of the cell membrane using ionophores and blockade of the Na^+^/K^+^ ATPase, solutions with of different K^+^ concentration were sequentially applied and the resulting fluorescence signal recorded (**Fig. 2C**). Satisfactory permeabilization was evaluated by the rapid establishment of plateau fluorescence values, that were then plotted against the known K^+^ concentration (**Fig. 2D**) to enable retrospective calculation of K^+^ concentration variations during the experiment. Under our experimental conditions and using this procedure, the resting [K^+^]_i_ was found to average 133±1 mM, which is consistent with expected mammalian cell [K^+^]_i_ values [20],[21],[27].

Taken together, one can conclude from this *in vitro* and intracellular characterization that APG-1 can be safely used as a K^+^-sensitive intracellular indicator.

### [K^+^]_o_ level influences the cytosolic K^+^ in astrocytes

**Figure 3:**
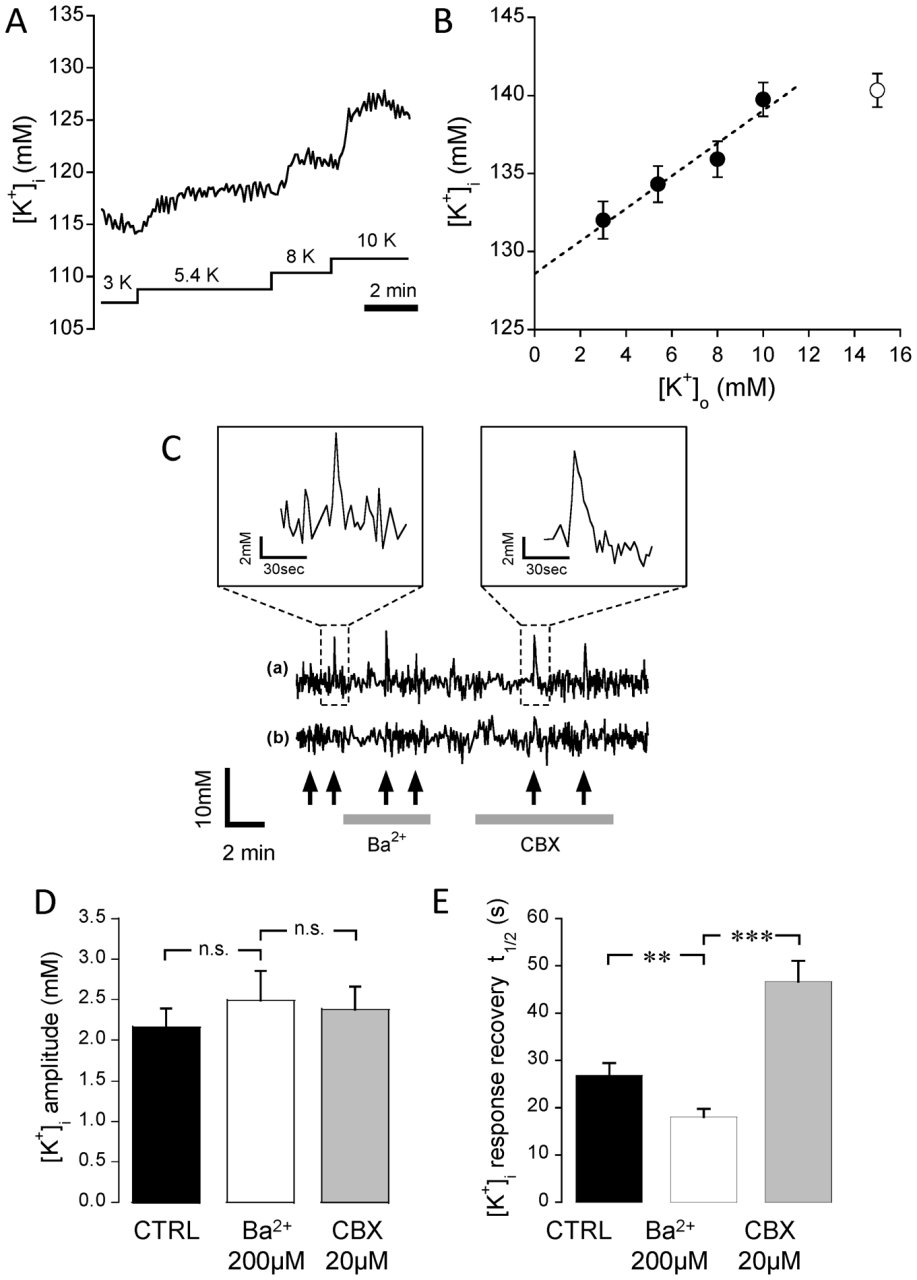
Intracellular K^+^ is modulated by [K^+^]_o_ level changes. (**A**) Representative single-cell [K^+^]_i_ trace during bath application of solutions with different K^+^ concentrations in the range 3 to 10 mM, as are found during physiological and pathological conditions. (**B**) Relationship between steady-state [K^+^]_i_ (measured on plateau levels) and externally applied [K^+^]_o_ (n = 120 cells from 12 exp). The graph indicates a steady increase in [K^+^]_i_ in the [K^+^]_o_ range 3–10 mM (plain circles), which yielded a slope of 1.04±0.06 (r = 0.82). A higher [K^+^]_o_ of 15 mM (open circle) failed to further increase [K^+^]_i_. (**C**) Intracellular K^+^ is influenced by localized K^+^-gluconate puff applications. Representative [K^+^]_i_ traces (average values of 7 cells each) during puff application (black arrows) of K^+^ gluconate in close proximity to the pipette (upper trace) and at>90 µm distance (lower trace). Insets: magnification of the trace after single extracellular applications of K^+^. Average amplitude (**D**) and duration of [K^+^]_i_ rise (**E**) induced by K^+^ puffs (black bar) compared with responses observed in the presence of 200 µM Ba^2+^ (white bar) or 20 µM carbenoxolone (CBX, grey bar) (n = 62 cells, 5 exp). No significant changes in amplitudes were found, whereas the response duration was significantly prolonged by CBX and reduced by Ba^2+^.

Because astrocytes are major actors in the regulation of interstitial K^+^ in the brain [2], we asked to what extent [K^+^]_i_ is influenced by [K^+^]_o_. We therefore loaded primary astrocytes with APG-1 AM and applied different solutions containing K^+^ in the expected concentration range that these cells may be exposed to in the brain. **Fig. 3A&B** shows that changing [K^+^]_o_ reproducibly caused a change in [K^+^]_i_ levels that followed [K^+^]_o_ changes proportionally in the range 3–10 mM (slope = 1.04±0.06, n = 120 cells, 12 exp). No further increase in [K^+^]_i_ was observed for 15 mM [K^+^]_o_. In addition, the lower slope and the kinetics of the changes with an initial transient, is consistent with some degree of cell volume regulation.

To investigate whether the APG-1 can be used measure responses to rapid and spatially limited [K^+^]_o_ elevations on [K^+^]_i_, we applied single short extracellular puffs from a K^+^-gluconate-filled pipette (**Fig. 3C**). [K^+^]_i_ rose rapidly with an average amplitude of 2.2±0.2 mM (**Fig. 3D**) and recovered to half amplitude within 26.8±2.6 s (**Fig. 3E**) in close proximity to the pipette tip (upper trace), whereas no responses were found at distances >90 µM (lower trace). Application of 200 µM Ba^2+^ did not prevent the initial K^+^ influx, but it did decrease the duration of the response (**Fig. 3D&E**). The mechanism underlying the shortened response duration is currently unclear.

After taking up the transient local excess of [K^+^]_o_ occurring in the vicinity of activated neurons, astrocytes are thought to redistribute it through the gap junctions over the syncytium towards lower local [K^+^]_i_, from whence it eventually returns to the extracellular space [31]–[33]. We therefore investigated whether gap junctions were involved in shaping the observed [K^+^]_i_ response. In the presence of carbenoxolone (CBX, 20 µM), a blocker of gap junction [34], the half-time of recovery of [K^+^]_i_ doubled (46.7±4.3 s), whereas the amplitude was not significantly altered (2.4±0.3 mM) (**Fig. 3C–E**). This result is consistent with the notion of the spatial redistribution of K^+^ over the astrocytic syncytium as proposed previously [2], [35]. In situations of impaired by gap junction communication, K^+^ excess persists longer within single cells.

### Glutamate transporter activity dynamically influences [K^+^]_i_

**Figure 4:**
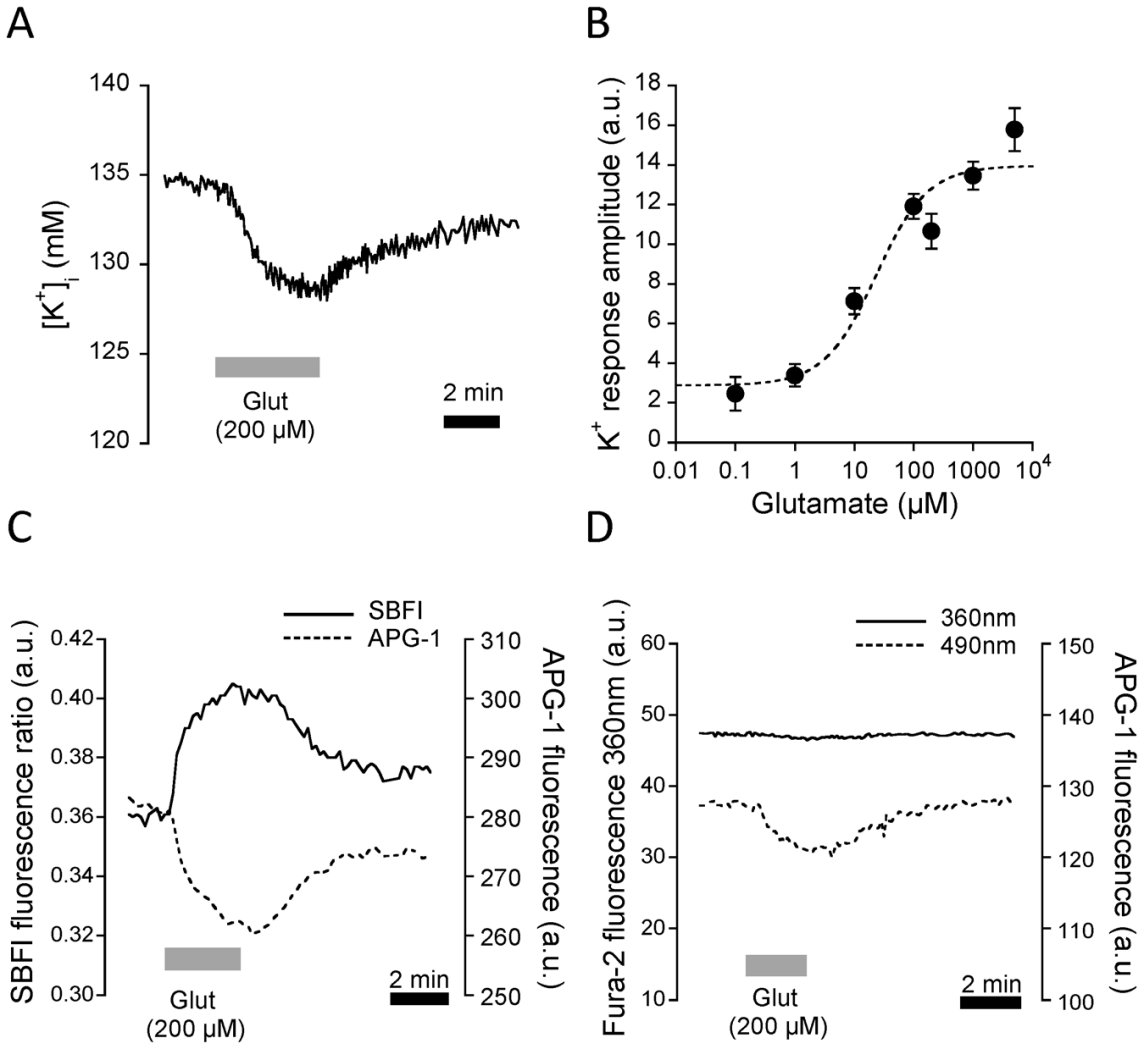
Intracellular K^+^ is modulated by glutamate application. (**A**) Application of 200 µM glutamate induced a rapid and reversible [K^+^]_i_ decrease with a amplitude of 2.3±0.1 mM (**B**) The [K^+^]_i_ response to glutamate application in the range 0.1 µM to 10 mM followed a Michaelis and Menten kinetics with an apparent EC_50_ of 11.1±1.4 µM. (**C**) Simultaneous Na^+^ and K^+^ monitoring using SBFI and APG-1, respectively. SBFI was sequentially excited at 340 and 380 nm, and its fluorescence excitation ratio computed, and APG-1 was excited at 490 nm. Glutamate application (200 µM) is indicated in the graph. Exemplar single cell trace out of three experiments. (**D**) The fluorescent dye Fura-2 co-loaded with APG-1 was excited at 360 nm, its Ca^2+^-insensitive wavelength, whereas APG-1 was excited at 490 nm. Whereas 200 µM glutamate application caused a decline of the APG-1 fluorescence response, that of Fura-2 at 360 nm remained unperturbed (Exemplar trace out of two experiments).

Glial high-affinity glutamate transporters have a complex stoichiometry in which glutamate influx is coupled with three Na^+^ ions and one proton in exchange with one K^+^ ion [10], [11]. Activation of this transporter is sufficient to induce substantial [Na^+^]_i_ changes in astrocytes both in culture and in situ [7], [23]. We therefore asked whether the K^+^ efflux associated with the glutamate transport cycle could alter [K^+^]_i_ levels in a detectable manner. **Fig. 4A** shows a single cell trace during application of glutamate, which caused a reversible decrease in [K^+^]_i_. The [K^+^]_i_ drop induced by 200 µM glutamate averaged 2.3±0.1 mM (n = 89 cells, 9 exp) for 200 µM glutamate application performed in 5.4 mM external K^+^ conditions.

In order to verify whether this [K^+^]_i_ response was glutamate-concentration dependent, we applied glutamate in the concentration range of 0.1 µM-10 mM, and measured the APG-1 fluorescence response. The amplitude of the K^+^ response was proportional to the bath concentration of glutamate and followed Michaelis and Menten kinetics (**Fig. 4B**). Non-linear curve fitting using equation [1] yielded an EC_50_ of 11.1±1.4 µM (n = 116 cells, 12 exp). Thus, both the kinetics exhibited by the K^+^ response to glutamate and the apparent affinity of the effect are consistent with a mediation of the effect by glutamate transporters. If this is the case, concurrent Na^+^ responses should be seen in cells undergoing a K^+^ response. We therefore loaded both the UV Na^+^ indicator SBFI and APG-1, which are spectrally compatible. Sequential illumination at 340 nm, 380 nm and 490 nm enabled recording the SBFI fluorescent ratio and the APG-1 fluorescence response, reflecting Na^+^ and K^+^ changes, respectively, in the same cell. **Fig. 4C** shows that astrocytes displayed opposite responses to glutamate, compatible with the activation of glutamate transporters causing both an increase in Na^+^ and a decrease in K^+^ with similar kinetics. In comparison, we loaded the UV probe Fura-2, and illuminated it at its isosbestic—*i.e.* Ca^2+^-insensitive— wavelength. Whereas APG-1 showed a downward fluorescence response to glutamate, Fura-2 at 360 nm yielded no fluorescence change in response to glutamate, indicating the absence of cell swelling that could have participated in the APG-1 fluorescence decrease.

## Discussion

Normal brain activity involves substantial movements of ions secondary to the generation of action potentials. In particular, neuronal excitation leads to elevations of extracellular K^+^ levels that have to be rapidly normalized in order to enable subsequent activity [3]. Of equal importance, concurrent excitatory neurotransmitter glutamate released from activated synapses, must be removed from the interstitial space to cope with high frequency synaptic transmission and prevent excitotoxic glutamate accumulation. Astrocytes play a central role in both these processes, which makes them essential players in the regulation of neuronal activity. In the present study, we employed a novel K^+^-sensitive fluorescent indicator to demonstrate that [K^+^]_i_ is significantly influenced by both external K^+^ and by glutamate transport activity.

Intracellular K^+^ measurements have been hampered until now by the lack of adequate fluorescent probe. The only one previously available, PBFI, suffers from a low quantum yield, a poor selectivity for K^+^ over Na^+^, an ultraviolet excitation, as well as an affinity for K^+^ of ∼8 mM [19] that renders it poorly sensitive to K^+^ in its physiological intracellular range of >100 mM. Moreover, the membrane permeant form of this dye is notoriously difficult to load in mammalian cells. For the present study, we used the recently synthesized K^+^ probe APG-1 and characterized its basic properties both in cuvette and *in situ*. We found that APG-1 generates important fluorescence changes in response to K^+^ concentration changes, and is not saturated at physiological intracellular K^+^ levels, *i.e.* above 100 mM. Contrary to PBFI (see e.g. [27]), APG-1 fluorescence is only marginally influenced by Na^+^ and by pH at their respective physiologically relevant intracellular levels. The spectral properties of APG-1 are very similar to those of the related Na^+^ indicator ANG-1 recently described [26]. As with this latter indicator, the membrane-permeant form of APG-1 readily loads into astrocytes and yields a bright and evenly distributed fluorescence, indicating a predominant cytosolic localization. Peak excitation and emission measured intracellularly were found to be red shifted by ∼10 nm compared with measurements performed in cuvettes. This bathochromic shift is frequently observed with fluorescent dyes, including fluorescein, when studied inside living cells, and reflects interactions with intracellular macromolecules [36].

Using this approach, we could measure detectable [K^+^]_i_ changes in register with bath K^+^ concentration changes in the range 3–10 mM [K^+^]_o_. This result is in line with previous measurements of [K^+^]_i_ performed using K^+^-sensitive microelectrodes (see e.g. [12], [16]). The fact that no further increase in [K^+^]_i_ was observed at 15 mM [K^+^]_o_ is indicative of the presence of a saturable pathway for K^+^ entry and/or a regulatory mechanism. In order to determine whether APG-1 could be used to investigate [K^+^]_i_ responses to local transient increases in [K^+^]_o_ we applied puffs of K^+^ from a pipette positioned close to target cells in the field of view. Indeed, such local puffs induced a short-lived but detectable intracellular response consisting in an initial sharp [K^+^]_i_ rise followed by a slower recovery to baseline. Cells at a distance of >90 µm from the pipette tip did not exhibit a change in [K^+^]_i_. In these experiments, application of Ba^2+^ did not inhibit the fast [K^+^]_i_ response, indicating that K_ir_ channels were not likely to be directly involved. The exact role and involvement of K_ir_ channels in K^+^ buffering remains a matter of debate [6], [37], [38] and the repertoire of K^+^ conductances underlying the passive conductance of K^+^ in astrocytes remains uncertain [4], [39]. The fact that Ba^2+^ did not abolish the [K^+^]_i_ response, likely indicates that other mechanisms, possibly Na/K/2Cl co-transporters (NKCC1) or the Na^+^/K^+^ ATPase, are involved, as previously proposed [6]. Members of the two-pore domain K^+^ channel family, namely TWIK-1 and TREK-1, have been recently shown to be expressed and functional in astrocytes [39]. Since these channels are resistant to Ba^2+^, they could represent an alternative candidate mediating the observed response. In these experiments, the contribution of the astrocytic syncytium in quickly dispersing the increased [K^+^]_i_ was demonstrated by blocking the gap junctions, which markedly increased the duration of the [K^+^]_i_ elevation in the stimulated cell.

Here we also demonstrate for the first time, to the best of our knowledge, that [K^+^]_i_ levels in astrocytes are decreased by the application of glutamate. The observed [K^+^]_i_ drop is attributable to the activity of glutamate transporters whose transport cycle involves the exchange of one K^+^ ion transported out of the cell, with three Na^+^ ions and a proton that are co-transported with glutamate into the cell [9], [11]. It should be pointed out that the contribution of ionotropic glutamate receptors in the response is unlikely since the application of NMDA does not evoke responses in the primary astrocyte model used in this study [28]. We also previously showed that AMPA receptor desensitization in primary astrocytes prevents a sizable cation influx that influences intracellular concentrations [23]. The kinetics of the observed response, the simultaneously measured Na^+^ rise, and the apparent EC_50_ of the response to glutamate of 11 µM are all identical to that published earlier in similar experimental conditions when measuring the glutamate transport associated Na^+^ response in astrocytes [23]. It is worth noting that resting extracellular glutamate is maintained in the low micromolar range. During neuronal activity it has been estimated that glutamate reaches the astrocyte membrane with a concentration that ranges from 10 µM [40] to 160–190 µM [41]. Our results obtained using primary astrocytes indicate that APG-1 is sensitive enough to detect K^+^ changes occurring within the physiological concentration range of glutamate. Whether this is also the case in brain slice astrocytes requires further experimental validation. Given that there should be a ratio of 3:1 of Na^+^ versus K^+^ movement during each transport cycle, one could postulate that the K^+^ response should be three-fold lower than the Na^+^ response. However, we observed an amplitude of [K^+^]_i_ response to glutamate of only 2.3 mM, i.e. about 5–10-fold lower than that of [Na^+^]_i_ under similar experimental conditions [see e.g. 23]. Therefore, it appears that a concurrent K^+^ influx during glutamate application may be rapidly activated to compensate for the cytosolic K^+^ drop. One candidate could be the Na^+^/K^+^ ATPase which undergoes rapid activation during glutamate uptake in astrocytes [23]. While the Na^+^/K^+^ ATPase pumps two K^+^ ions into the cell for 3 Na^+^ that are extruded, glutamate transport exchanges one K^+^ for three Na^+^. Thus, if one considers the Na^+^/K^+^ ATPase and glutamate transport as a functionally coupled complex, as has been suggested [42]–[44], during glutamate transport, overall the K^+^ influx would be larger than its efflux, possibly explaining the smaller than expected decrease in [K^+^]_i_ during glutamate transport. Another alternative could be a possible involvement of mitochondria that have been proposed to sequester K^+^ in astrocytes [21]. Mitochondria have been shown to be actively involved during astrocytic responses to glutamate, in particular by taking up Na^+^ [45] and protons [28] into their matrix. It is conceivable that under these conditions mitochondria, through their ability to take up and release K^+^ may act as intracellular K^+^ buffers and thus reduce the [K^+^]_i_ changes. This idea could explain both the K^+^ drop during glutamate transport and the slope of [K^+^]_i_ change upon external K^+^ level changes, that were found to be lower than anticipated in the present study.

In conclusion, this study demonstrates that the novel fluorescent K^+^ indicator APG-1 is a powerful new tool that can be employed to address detailed and fundamental questions about the regulation of [K^+^]_i_. Of particular relevance is our novel finding that glutamate uptake by astrocytes has a significant influence on [K^+^]_i_, a finding that represents an important contribution to an integrated understanding of astrocyte functions in the brain.

## Supporting Information

Figure S1 **Spectrofluorimetric characterization of the influence of Na^+^ on APG-1 fluorescence.** Fluorescence emission plotted as a function of Na^+^ concentration in intracellular-like solution containing increasing concentrations of K^+^ (0–150 mM). The total monovalent cation concentration was kept constant by appropriate additions of NMDG. Each curve represents triplicate measurements.
(TIF)
